# Testing turnover intentions and organizational justice on servant leadership: a validation of the servant leadership survey scale in sub-Saharan Africa

**DOI:** 10.1108/LHS-05-2024-0048

**Published:** 2025-03-04

**Authors:** Simon Nantamu, Adele Grazi, Sam Cromie

**Affiliations:** School of Psychology, Makerere University, Kampala, Uganda, and; Centre of Innovative Human Systems, School of Psychology, Trinity College Dublin, Dublin, Ireland

**Keywords:** Servant Leadership, Employee turnover intentions, Organizational justice, Health care, sub-Saharan region, Uganda

## Abstract

**Purpose:**

Servant leadership, a form of holistic leadership, has been primarily explored in Western cultures. Consequently, there is a lack of understanding on whether servant leadership style is transferable to other cultures, posing a scientific but also ethical challenge in the leadership literature. The purpose of this study is to validate a servant leadership scale (Van Dierendonck and Nuijten, 2011) in the sub-Saharan region (specifically in Uganda) in the health care context. Furthermore, with the aim of improving quality of patient care, it explores the impact servant leadership has on organizational justice and employees’ turnover intentions.

**Design/methodology/approach:**

The cross-sectional study gathered data from 13 public Regional Referral Hospitals in Uganda. After ethical approval, self-administered surveys were distributed in the 13 survey locations. The survey used standardized scales to measure servant leadership, organizational justice and employee turnover intentions. A total of 355 respondents completed the survey.

**Findings:**

Results of the confirmatory factor analysis supported six of the eight factors of the servant leadership scale. Furthermore, regression analysis showed a significant relation between servant leadership and organizational justice (*r* = 0.678; *p* < 0.01) and a negative relation between servant leadership and employee turnover intentions (*r* = −0.139; *p* < 0.01).

**Originality/value:**

This study brings an important contribution to the functionality of the servant leadership scale in a non-Western context. It also provides insight into the positive impact servant leadership style can have on health workers and patient care.

## Introduction

Servant leadership has been considered an approach to leadership with global applicability (Neubert *et al.*, 2008). Nevertheless, servant leadership has mainly been studied in Western contexts and up to date research exploring servant leadership in other cultures has been limited and largely qualitative warning the generalizability and comparability of findings (Koshal, 2005; Irving and McIntosh, 2006). Servant leadership has particular promise for study of leadership in sub-Saharan Africa as it would seem to be in tune with African concepts of leadership and community (Ncube, 2001). Indeed, it could meet the need identified by Lutz (2009) for a theory of management consistent with African traditional cultures. Servant leadership has been proposed as a particularly suitable leadership model for healthcare because it focuses on the strength of the team, developing trust and serving the needs of patients (Aij and Rapsaniotis, 2017; Gunnarsdóttir *et al.*, 2018). If servant leadership is meaningful as a leadership approach in sub-Saharan Africa, it needs to be demonstrated to be predictive of key measures of organizational climate, such as organizational justice and turnover intentions.

This paper presents a validation of a standardized measure of servant leadership in the Ugandan health care sector together with an investigation of its predictive validity for organizational justice and turnover intentions.

## Defining servant leadership

Servant leadership as a theoretical framework was originally advanced by Greenleaf (1977) and is characterized by strong follower-centric, altruistic, moral/ethical and spiritual values (Jaworski, 1997; Latif and Marimon, 2019; Smith *et al.*, 2004). Under this theoretical framework, followers become the primary concern for servant leaders that go beyond self-interest and organizational goals (Block, 1993; Hale and Fields, 2007).

Greenleaf (1977, pp. 13–14) defined servant leadership as leadership that empowers followers to “grow healthier, wiser, freer, more autonomous and more likely themselves to become servants”. Servant leaders put stewardship at the core of their leadership values, and they recognize the importance of serving others, particularly employees who might be marginalized by the organizational system. Core to servant leadership is not only the desire to serve others first but also to enhance employees’ wellbeing by showing genuine care toward employees’ needs. This is generally achieved by servant leaders through a strong developed sense of ethics, integrity, morality and trustworthiness (Parris and Peachey, 2013).

## Relevance of servant leadership in the sub-Saharan Africa context

Servant leadership theory has been primarily developed in the context of high-income settings and individualistic societies, generally associated with Western culture (Brubaker, 2013; Curry, 2012; Hale and Fields, 2007; Stein *et al.*, 2020). Applying these theories on a worldwide basis might not be appropriate, especially in cultures, which differ significantly from Western cultures. Table 1 highlights core cultural differences between sub-Saharan Africa and Western societies (Pietersen, 2005).

**Table 1.** Comparison of sub-Saharan Africa and Western core cultural values

	Sub-Saharan Africa values	Western values
1	Ethnocentrism	Eurocentrism
2	Collectivism/communalism	Individualism
3	Traditionalism	Modernity
4	Humanistic orientation	Instrumental orientation
5	Group solidarity	Rationality
6	Conformity to norms	Independence

**Sources:** Author’s own work; Pietersen (2005)

Evidence suggests that leadership is deeply rooted to culture as both the leader’s actions and followers’ responses inevitably reflect forms of behavior which is regarded as legitimate and appropriate within their society (Dickson *et al.*, 2012; Hanges *et al.*, 2016; Segundo-Marcos *et al.*, 2022). The replication of Western management and leadership in non-Western environments has been a contested issue in the management literature (Ahiauzu, 1986; Montgomery, 1985). However, the global leadership and organizational behavior effectiveness research (GLOBE) have conceptualized servant leadership as a humane and holistic orientation that can be applied throughout the world, suggesting therefore that servant leadership is a global leadership style (Ezeorah, 2023). Indeed, the model of servant leadership has been explored in non-Western cultures such as Africa, Asia and Latin America, nonetheless the majority of these studies have explored servant leadership through a qualitative design, which do not produce generalizable findings (Koshal, 2005; Irving and McIntosh, 2006). Moreover, within the African context, servant leadership has only been explored in South Africa and Ghana with poor focus on how this leadership style can benefit the healthcare system (Manala, 2014; Swart *et al.*, 2021; Stouraitis *et al.*, 2021).

African leadership generally appears to align to the Ubuntu philosophical system that “holds promise for progressive and ethical change for Africa” (Ncube, 2001, p. 77). Ubuntu can be translated into “I am because we are” or “humanity”, which is a cultural view held by the Bantu tribes of Africa which strives to reduce the African plague of genocide, corruption, authoritarian leadership and human suffering by emphasizing the interconnectedness of ethical practices between individuals and society (Le Grange, 2011; Murithi, 2009). According to Wanasika et al (2011), the humane orientation clearly plays a significant role in outstanding leadership behavior in sub-Sahara countries, it permeates the core of societal and organizational life in sub-Sahara Africa.

African managers, therefore (Lutz, 2009) need a management theory consistent with their communal cultures, not only in the interest of moral integrity and social stability, but also in the interest of economic productivity. Servant leadership aligns with the humanistic Ubuntu philosophy of the SSA region and an exploration of how such leadership style impacts the healthcare system is considered of relevant importance, as not only are health care workers under extreme work pressure but are also exposed to very vulnerable people.

## Servant leadership and health care

Health care institutions seek a leadership style that supports the wellbeing of health care workers and quality of care for their patients. Leadership is focal to the functioning of health care systems as suggested by the World Health Organization (Curry, 2012) and the African Working Group of the Joint Learning Initiative (2006).

Various scholars (e.g. Aij and Rapsaniotis, 2017; Gunnarsdóttir *et al.*, 2018; Hanse *et al.*, 2015; Koh, 2009) agree that servant leadership is a promising leadership model for the delivery of patient centered and high value care. Not only could servant leadership improve the quality and efficiency of health work providers but could also reduce organizational costs (i.e. employee turnover). Moreover, such leadership style is shown to create a more human organizational culture that not only impacts staff wellbeing but also patients care.

In Africa, the underdevelopment of the health care systems and lack of funding urge for radical solutions. Improving the quality of care might represent a way to reduce costs, reduce medical errors and delays in health delivery and efficiency (Oleribe et al, 2019). Specifically, increased investment in leadership capacity has been urged in SSA to increase healthcare workers motivation and productivity to mitigate the lack of minimum wage policies (Ssengooba *et al.*, 2005).

Specifically in Uganda, the context of present research, health services are provided by both the public and private subsector with each subsector covering about 50% of the reported output. Public health services in Uganda are delivered through Health Centre IIs, Health Centre IIIs, Health Centre IVs, General Hospitals, Regional Referral Hospitals (RRHs) and National Referral Hospitals (NRHs). The range of health services delivered varies with the level of care; the RRHs and NRHs are semiautonomous supervised by the Ministry of Health, whereas General Hospitals and lower-level facilities are supervised by district local governments. Current contribution to total health expenditure is 25.6% by government, 29% from out-of-pocket (OOP) and 45.4% by development partners (Ministry of Health, 2023). Uganda is experiencing a serious human resources crisis in the health sector and understanding how to reduce costs within the disposed budget is crucial for the effectiveness of these services.

## Conceptualizing and development of servant leadership scale

Building on Greenleaf (1977), Spears (1995, 1998, 2002, 2010) brought forward an important contribution by identifying ten characteristics of servant leadership. Later Patterson (2003, *p* 81) who defined servant leadership as “those who serve with a focus on the followers, whereby the followers are the primary concern and the organizational concerns are peripheral”, proposed 7 components or values that shape the behavioral attitudes of a servant leader. In 2008, Sendjaya, Sarros and Santora (2008) developed six domains of servant leadership. This brought an important contribution to servant leadership theory as they highlighted for the first time that servant leaders should view themselves as stewards who encourage their followers to achieve their full potential.

More recently, van Dierendonck and Nuijten (2011) developed an eight components model of servant leadership, derived from an analysis and synthesis of available literature. Importantly, van Dierendonck and Nuijten (2011) postulated that servant leaders are essential to providing direction to followers and claim that while previous scales focused on the “follower” side of servant leadership, the servant leadership survey (SLS) incorporates both the “leader” aspects and the followers aspect and measured the leader–follower relationship from the perspective of the follower (Van Dierendonck and Nuijten, 2011). Furthermore, apart from including the essential domains of servant leadership, the SLS validation studies have demonstrated to have factorial validity, internal consistency, content validity, incremental validity and criterion-related validity (Van Dierendonck and Nuijten, 2011). There are other studies that have also supported the validity of the SLS (van Direndock *et al.*, 2017; Kobayashi et al, 2020). We therefore argue that the SLS should be considered the benchmark measure of servant leadership. Table 2 presents an overview of some of the main servant leadership models.

**Table 2.** Diverse conceptualizations of servant leadership

Spears (1995) 10 components of servant leadership	Patterson (2003) 7 components of servant leadership	Sendjava *et al.* (2005) 6 components of servant leadership	Van Dierendonck and Nuijten, 20118 components of servant leadership
Listening	Agápao love	Voluntary subordination	Stewardship
Empathy	Humility	Authentic self	Courage
Healing	Altruism	Covenantal relationship	Humility
Awareness	Vision	Responsible morality	Authenticity
Persuasion	Trust	Transcendental spirituality	Empowerment
Conceptualization	Empowerment	Transforming influence	Forgiveness
Foresight	Service		Standing back
Stewardship			Accountability
Helping people grow			
Building community			

**Source:** Author’s own work

Van Dierendonck and Nuijten (2011) proposed a definition of the key servant leadership characteristics based on combined insights of the most influential theoretical models and conceptualizations of servant leadership in the leadership literature (Greenleaf, 1977; Spears, 1995; Laub, 1999; Russell and Gregory Stone, 2002; Liden *et al.*, 2008). The SLS includes the following eight dimensions: empowerment, accountability, standing back, humility, authenticity, courage, interpersonal acceptance or forgiveness and stewardship.

*Empowerment* is defined as a motivational concept focused on enabling people and encouraging personal development (Conger, 2000). *Accountability* refers to holding people accountable for performance they can control (Conger, 1989). *Standing back* is the extent to which a leader gives priority to the interest of others first and gives them the necessary support and credit (van Direndock *et al.*, 2017). *Humility* focuses on daring to admit that one is not infallible and does make mistakes (Morris *et al.*, 2005). *Authenticity* is closely related to expressing the “true self”, expressing oneself in ways that are consistent with inner thoughts and feelings (Harter, 2002). *Courage* refers to daring to take risks and trying out new approaches to old problems (Greenleaf, 1991). *Interpersonal acceptance/forgiveness* refers to the ability to understand and experience the feelings of others, understand where people come from (George, 2000), and the ability to let go of perceived wrong doings and not carry a grudge into other situations (McCullough *et al.*, 2000). Finally, *stewardship* is the willingness to take responsibility for the larger institution and go for service instead of control and self-interest (Block, 1993; Peterson and Seligman, 2004).

Van Direndonck and Nuijten (2011) developed the SLS using a European sample (Netherland and UK). Therefore, there is the need to further examine the external validity of the SLS through replication studies in both developed and developing countries (Latif and Marimon, 2019). The present research contributes to existing research by further validating SLS in the sub-Saharan Africa (H 1).

## Servant leadership and turnover intentions

Turnover intention refers to the subjective evaluation by a person regarding the likelihood of leaving their employer in the immediate future (Mowday *et al.*, 1982). Self-reported turnover intention has been confirmed to predict eventual turnover (Tekleab *et al.*, 2005). Accordingly, turnover intention is widely used as a proxy measure of actual turnover within organizational settings (Harris *et al.*, 2005). High staff turnover negatively affects health care by increasing workload, undermining team morale, creating disruptions and inefficiencies in work processes and causing a loss of institutional knowledge (World Health Organization, 2006).

Research indicates that servant leadership reduces turnover intentions through the moderating and mediating effect of several work constructs such as: perceived organizational support, job satisfaction, job embeddedness, affective organizational commitment and organizational commitment (Huning *et al.*, 2020). Nevertheless, because servant leadership is posited to be the highest commitment of leaders to their followers, it should be a model that ameliorates employee turnover and turnover intentions (Jaramillo *et al.*, 2000; Liden *et al.*, 2008). Based on this argument, we test the relationship between servant leadership and employee turnover intentions, expecting servant leaders to reduce employees’ turnover intentions.

## Servant leadership and organizational justice

Empirical evidence shows that a positive association between servant leadership and justice perceptions exists (Mayer *et al.*, 2008; Ehrhart, 2004), which comes as no surprise. Servant leaders are characterized by strong follower-centric, altruistic, moral/ethical and spiritual values which makes it likely that they treat their followers with fairness, creating a climate of organizational justice (Mayer *et al.*, 2008).

Research in organizational justice has typically not focused on leaders as sources of justice with the exception of the more recent focus on interactional justice (van Knippenberg *et al.*, 2007). Rather, it has focused on more systemic or institutionalized forms of justice such as distributive and procedural justice. In contrast, research integrating leadership and justice argue that employees’ perception of leaders’ fairness should also be explored, as leadership has a direct influence on justice perceptions and that justice is a means by which leader effectiveness is actualized (Colquitt, 2001).

Mayer *et al.* (2008) argued that servant leaders will treat employees in an interpersonally sensitive manner thus improving followers’ sense of justice. Servant leaders’ moral orientation is likely to help employees engage in ethical behaviors and reduce bias from decision making processes. Further, servant leaders provide an opportunity for followers to voice their concerns which can lead to higher trust in leader and perceptions of fairness (Mayer *et al.*, 2008). Finally, servant leaders focus on the growth and development of followers, thus improving justice perceptions (Mackasare, 2022).

In sum, existing empirical research has poorly examined how servant leadership is associated with and predicts organizational justice, making this an important contribution of the study (Hanh and Choi, 2019; Mayer *et al.*, 2008). Therefore, we test whether servant leadership is positively associated with organizational justice.

## Method

### Participants

The study used a cross-sectional design. The sample of this study was health workers working across 13 public RRHs in Uganda. To partake in the survey respondents had to have worked for at least nine months under their current immediate supervisor at the public RRHs on full-time and permanent appointment by the Government of Uganda. The minimum professional qualification for inclusion was a certificate in one’s discipline of specialization from an institution within or outside sub-Saharan Region. Within the data collection period, 355 completed questionnaires were received which is representative of the estimated population of 2600 healthcare workers based on the table for sample determination (Krecjie and Morgan, 1970). The data was collected from RRHs in Uganda which are in between the National Referral Hospitals and the District governed health facilities; there is hardly any data published in which human resources for health information at the RRHS is disaggregated demographically. Only questionnaires that were fully completed with no missing data are reported.

The sample consisted of nurses/midwives: 46.8% (*n* = 166), doctors: 4.2% (*n* = 15), clinical officers: 17.2% (*n* = 61) and other healthcare workers (pharmacy/dispensary, anesthetic and laboratory staff): 31.8% (*n* = 113). Male respondents constituted 51.8% (184) and female respondents constituted 48.2% (171). Age groups of respondents were: 1.1% (4) under 25 years, 12.7% (45) between 25 and 29 years, 36.1% (128) between 30 and 39 years, 33.2% (118) between 40 and 49 years and 16.6% (59) were over 50 years. Time in service of participants:13.2% (47) under 5 years, 20.3% (72) between 5 and nine years, 22% (78) between 10 and 14 years, 15.2% (54) between 15 and 19 years, 11.0% (39) between 20 and 25 years and 18.3% (65) over 25 years. Immediate supervisors were 48.2% (171) male and 51.5% (183) female.

In the health audit by the Ministry of Health undertaken in Uganda in 2017, nurses/midwives were reported to make up 69.3% of the health workers, 10.7% were clinical officers, 6.0% were doctors and 14.1% was made up of other health workers.

### Procedure

After ethical approvals was granted, permission to administer the questionnaire in the hospitals was obtained by Uganda’s Ministry of Health. Thereafter, permission was also granted by hospital directors of the 13 RRHs. On obtaining the above clearance, a research assistant in each of the 13 study sites was recruited to help with data collection. The assistants were duly oriented in the study protocol and administration of the questionnaire. Research assistants explained (verbally) to participants the aims of the research and issued an information leaflet. Respondents who agreed to participate in the study were provided with a paper and pencil, consent form and questionnaire. The completed questionnaires were retrieved after 2 weeks. Questionnaires were dispatched to the research assistants in the survey locations either physically or by post in sealed envelopes. And similarly, the completed questionnaires were delivered back to the researcher either physically or by sealed posted envelopes.

### Measures

*Servant leadership* was assessed using SLS (Van Dierendonck and Nuijten, 2011). It is made up of 30 items from eight subscales, namely, empowerment (7 items), standing back (3 items), accountability (3 items), forgiveness (3 items), courage (2 items), authenticity (4 items), humility (5 items) and stewardship (3 items). A 5-point Likert-type scale with scores ranging from 1 (very often) to 5 (rarely) was used for this instrument. A sample item is “My supervisor emphasizes the societal responsibility of our work.” The combined sample of all three studies undertaken during development of this measure demonstrated alpha coefficients of 0.89 for empowerment, 0.81 for accountability, 0.76 for standing back, 0.91 for humility,0.82 for authenticity, 0.69 for courage, 0.72 for forgiveness and 0.74 for stewardship. In this present study, the alpha coefficient for the 17 items that were retained was 0.88. The servant leadership domains that were retained after the confirmatory factor analysis (CFA) displayed the following alpha coefficients: empowerment (0.73), humility (0.86), accountability (0.77), stewardship (0.84), courage (0.70) and authenticity (0.62).

*Organizational justice* was measured using the organizational justice scale developed by Colquitt (2001). The scale comprises 20 items divided into four subscales:

1. procedural justice (7 items);
2. distributive justice (4 items);
3. interpersonal justice (4 items); and
4. informational justice (5 items).

A 5-point Likert-type scale with scores ranging from 1 (Very often) to 5 (Rarely) was used for this instrument. A sample item is “My performance outcomes controlled by my immediate supervisor reflect the effort I put into your work.” Previous study has indicated alpha coefficients of 0.96 (Padmakumari, 2013). In this study, this measure demonstrated an alpha coefficient of 0.95.

*Turn over intentions* was measured using 3 items from Walsh *et al.* (1985) and 3 items picked from Roodt (2004). A 7-point Likert with scores ranging from 1 (strongly disagree) to 7 (strongly agree) was used. The alpha coefficient retained in this current study was 0.88. An item sample “As soon as I find a better job, I will leave this job.” Walsh *et al.* (1985) reported Cronbach’s alpha of 0.90, whereas Bothman and Roodt (2013) reported a Cronbach’s alpha of 0.80 using 6 adapted items from Roodt’s scale (2004). In this study, this scale displayed an alpha coefficient of 0.88.

### Analytical strategy

Data collected was coded, entered, scored and analyzed in SPSS, version 22.0 (IBM Corp, 2010). The data was screened and cleared of errors; then tested for critical assumptions, namely: normality, linearity, homoscedasticity, outliers, multicollinearity and singularity (Tabachnick and Fidell, 2007; Pallant, 2005). Generally, the data met the required standards to proceed with CFA, correlations and regression analysis.

CFA was used to fit the theoretical model of servant leadership. The CFA used maximum likelihood (ML) estimation procedure to assess the model. The present research assessed 5 goodness of fit parameters: chi-square test, comparative fit index (CFI), the goodness of fit index (GFI), the root mean square error estimation approximation (RMSEA), standardized root mean square residual (SRMR). For the model to be acceptable, the following threshold values were used (Kline, 2011): *X*2 (> 0.05), CFI (≥ 0.90), GFI (≥ 0.90), RMSEA (≤ 0.08) and SRMR (≤ 0.10).

The revised measure of servant leadership and its domains were correlated with organizational justice and turnover intentions using Pearson product moment correlation coefficient (PPMCC) to test *H2* and *H3*. The revised overall measure of servant leadership was further used to predict organizational justice and turnover intentions using simple linear regressions, whereas the domains of servant leadership were used to predict organizational justice and turnover intentions using multiple linear regressions.

## Results

### Means, standard deviations and correlations of study variables

Means, standard deviations and intercorrelations of the eight-factor servant leadership model and two dependent variables (turnover intentions and organizational justice) are presented in Table 3. All the factors of servant leadership display significant positive correlation except for courage and accountability. Organizational justice shows a significant positive correlation with the resulting six-factor model and turnover intentions. Employee turnover intentions shows a significant negative relation with all six factors in the retained model expect for accountability and courage and a significant negative correlation with organizational justice.

**Table 3.** Means, standard deviations and intercorrelations of the eight-factor servant leadership model and two dependent variables (turnover intentions and organizational justice)

Eight-factor constructs	Mean	*SD*	1	2	3	4	5	6	7	8	9
Empowerment-1	3.668	0.851	1.000								
Humility-2	3.386	0.896	0.586**	1.000							
Accountability-3	3.884	0.722	0.438**	0.368**	1.000						
Stewardship-4	3.746	0.849	0.599**	0.635**	0.381**	1.000					
Courage-5	2.959	0.930	0.224**	0.189**	0.075	0.195**	1.000				
Authenticity-6	3.298	0.820	0.454**	0.459**	0.276**	0.454**	0.287**	1.000			
Servant leadership-7	3.454	0.576	0.671**	0.783**	0.581**	0.780**	0.540**	0.723**	1.000		
Organizational justice-8	3.552	0.729	0.623**	0.624**	0.324**	0.665**	0.223**	0.471**	0.678**	1.000	
Turn over intentions-9	2.559	1.280	−0.146**	−0.157**	−0.097	−0.175**	0.060	−0.116*	−0.139**	−0.134*	1.000

Notes:

*n* = 355; **p < 0.01; *p < 0.05

**Source:** Author’s own work

Furthermore, Table 4 provides a comparison between reliability coefficients of six of the eight factors of the servant leadership scale between the original and SSA samples. In the current study, six of the compound domains show acceptable reliability. The alpha for authenticity (0.62) may be considered low; however, it is not unacceptable (George and Mallery, 2003); and given that this research focuses on development and validation of a measure keeping the authenticity domain leaves the opportunity for further refinement.

**Table 4.** Comparison of reliability coefficients for the original study population and the sub-Saharan samples

Servant leadership domain	Original population		Sub-Saharan population	
	No. of items	Reliability coefficient	No. of items	Reliability coefficient
Empowerment	7	0.89	3	0.73
Accountability	3	0.81	3	0.77
Courage	2	0.69	2	0.70
Authenticity	4	0.82	3	0.62
Humility	5	0.91	3	0.82
Stewardship	3	0.74	3	0.84

**Source:** Author’s own work

### Confirmatory factor analysis of the servant leadership scale

**Figure 1.**
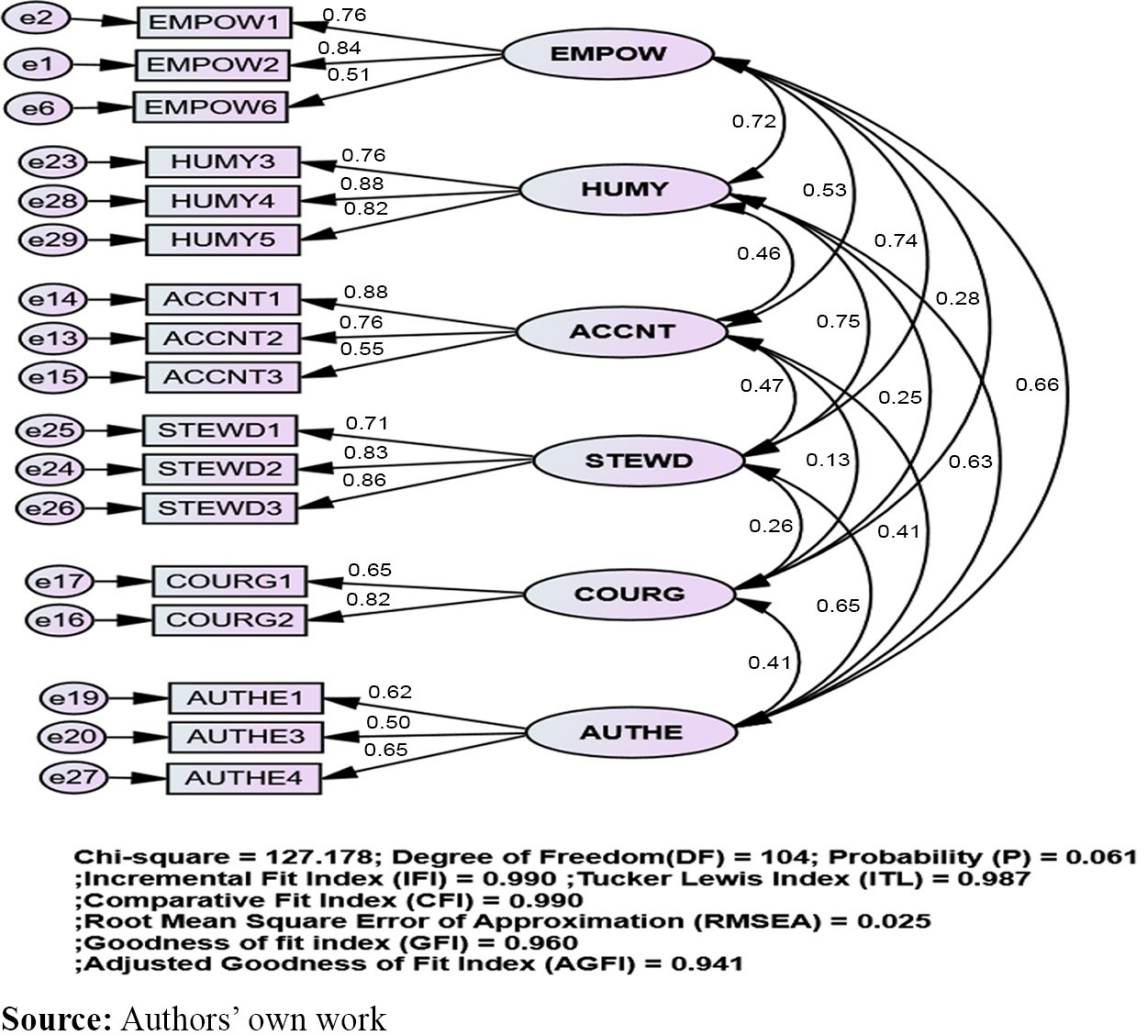
CFA of servant leadership eight-factor model

To validate the servant leadership scale (Van Dierendonck and Nuijten, 2011), a CFA was run. The CFA did not support all the eight-factor theoretical model (partially rejecting *H1*). Incremental removal of 13 items across the eight factors provided a six-factor model with 17 items with a good fit with the data (*X*^2^ = 127.18, *df* = 104, *p*< 0.06). Analysis of the fit indices show adequate fit: IFI (0.99), CFI (0.99), GFI (0.96), AGFI (0.94) and TLI (0.99). Finally, the RMSEA of 0.03 and SRMR of 0.06 indicate a good fit (Figure 1). Standing back and forgiveness were removed to attain the model fit (Li, 2015) with the guidance of modification indices in the AMOS 23.

### Regression analysis of servant leadership and organizational justice

The model summary and ANOVA results generated through simple liner regression showed overall servant leadership to be a significant predictor accounting for 46% of organizational justice (adjusted *R*^2^ = 0.46; *F* = 300.30; Sig. *F* = 0.00) (supporting *H2*) (Table 5).

**Table 5.** Regression analysis of servant leadership on organizational justice

	Unstandardized coefficients		Standardized coefficients			Collinearity statistics
Variables	B	Std. error	Beta	t	Sig.	VIF
(Constant)	0.587	0.173		3.384	0.001	
Servant leadership	0.858	0.050	0.678	17.329	0.000	1.000

Note:

Dependent variable = organizational justice

**Source:** Author’s own work

When multiple linear regression was run using all the six domains of servant leadership as predictors of organizational justice, the model summary indicated that there was significant prediction (adjusted *R*^2^ = 0.56; *F* = 74,56; Sig. *F* = 0.00) accounting for 56% of organizational justice. However, inspection of the *t*-values in confirmed only four domains of servant leadership to be significant predictors of organizational justice including empowerment (*t* = 5.09; Sig. = 0.00), humility (*t* = 4.53; Sig. = 0.00), stewardship (*t* = 6.54; Sig. = 0.00) and authenticity (*t* = 2.39; Sig. 0.02) (Table 6). The results show accountability and courage to be nonsignificant predictors.

**Table 6.** Multiple linear regression of six-factor servant leadership model on organizational justice

	Unstandardized coefficients		Standardized coefficients	t	Sig.	Collinearity statistics
	B	Std. error	Beta			VIF
(Constant)	0.809	0.171		4.731	0.000	
Empowerment	0.215	0.042	0.251	5.089	0.000	1.938
Humility	0.183	0.040	0.225	4.534	0.000	1.959
Accountability	0.025	0.041	0.024	0.607	0.544	1.289
Stewardship	0.282	0.043	0.328	6.541	0.000	2.001
Courage	0.026	0.029	0.033	0.880	0.379	1.107
Authenticity	0.091	0.038	0.102	2.387	0.018	1.451

Note:

Dependent variable = organizational justice

**Source:** Author’s own work

### Regression analysis of servant leadership and turnover intentions

The model summary and ANOVA results generated through simple liner regression showed overall servant leadership to be a significant predictor accounting for 2% of turnover intentions (Adjusted *R*^2^ = 0.02; *F* = 6.95; Sig. *F* = 0.01) (supporting *H3*; Table 7).

**Table 7.** Regression analysis of servant leadership on turnover intentions

	Unstandardized coefficients		Standardized coefficients			Collinearity statistics
	B	Std. error	Beta	t	Sig.	VIF
(Constant)	3.626	0.410		8.836	0.000	
Servant leadership	−0.309	0.117	−0.139	−2.635	0.009	1.000

Note:

Dependent variable = turnover intentions

**Source:** Author’s own work

However, results of the model summary of the multiple linear regression with the 4 domains of servant leadership that were significantly related to turnover intentions indicated the domains significantly predicted turnover intentions (*R*^2^ = 0.04; *F* = 3.26; Sig. *F* = 0.01). Inspection of the *t*-values showed that none of the four domains including empowerment (*t* = −0.55; Sig. = 0.59), humility (*t* = −0.77; Sig. = 0.45), stewardship (*t* = −1.43; Sig. = 0.16) and authenticity (*t* = −0.41; Sig. = 0.68) was a significant predictor of turnover intentions (Table 8).

**Table 8.** Multiple linear regression of six-factor servant leadership model on turnover intentions

	Unstandardized coefficients		Standardized coefficients			Collinearity statistics
Servant leadership factors	B	Std. error	Beta	t	Sig.	VIF
(*Constant*)	3.768	0.356		1.584	0.000	
Empowerment	−0.058	0.106	−0.039	−0.546	0.586	1.820
Humility	−0.080	0.105	−0.056	−0.765	0.445	1.945
Stewardship	−0.159	0.111	−0.105	−1.425	0.155	1.978
Authenticity	−0.040	0.097	−0.025	−0.412	0.681	1.392

Note:

Dependent variable = turnover intentions

**Source:** Author’s own work

## Discussion, implications and conclusion

The research makes several important theoretical contributions to the existing leadership literature. Servant leadership has been acclaimed as being an important leadership style that supports the wellbeing of quality-of-care workers (Curry, 2012). However, the majority of research to date has focused mainly in exploring the SLS in Western countries, which does not necessarily translate into other cultural contexts. Furthermore, the majority of research has been of qualitative nature, which cannot produce generalizable findings.

First, this research contributes to the existing leadership literature by validating the servant leadership scale (Van Dierendonck and Nuijten, 2011) within a sub-Saharan Context. Results confirmed a 17-item scale consisting of six of the original factors, except for standing back and forgiveness. These results show the construct validity of 6 of factors in SLS within the sub-Saharan region, which is an important empirical contribution. However, it is important to note that two factors forgiveness and standing back did not meet significance. Indeed, forgiveness has shown poor factor loading also in previous studies (Clark, 2019; Kobayashi *et al.*, 2020). It is argued that this might be due to the items being stated negatively, whereas items of other facets of the SLS are phrased positively. This study may be among the first to not confirm standing back as a facet of servant leadership. A possible explanation for this result is that servant leaders, give central stage to their followers but might not remain in the background as to not be considered cold, aloof and detached; suggesting that this may be result of how this factor has been operationalized rather than being context specific (Clark, 2019). Furthermore, previous studies (Gunnarsdóttir, 2014) have used what is referred to as the Icelandic version of the SLS in which the factor “standing back” was renamed as “servitude” which might indicate a conceptualization ambiguity, although the original items of the factor were maintained.

Second, the study validated the SLS not only in a sub-Saharan context but also within a healthcare context. Results show that overall, that servant leadership can be a useful leadership style to increase healthcare workers’ perceptions of workplace fairness and intentions to stay, although two factors might need to be revised.

Third, the study indicated that the six retained domains of SLS are significantly related to employee perceptions of organizational justice. These results imply that the six-servant leadership behaviors are relevant in the establishing a fair and just workplace environment in the SSA. However, the multiple linear regression analysis showed that only four of the domains of servant leadership (empowerment, humility, stewardship and authenticity) to be significant predictors of organizational justice. Accountability and courage have been shown to be nonsignificant predictors of organizational justice. These results may mean that empowerment, humility, stewardship and authenticity are the most meaningful and consequential servant leadership behaviors in nurturing workplace justice within the sub-Saharan Africa.

Fourth, the study indicates that the six retained domains of SLS are negatively and significantly associated with employee turnover intentions, suggesting that six-servant leadership behaviors enhance employee retention within healthcare systems in SSA. The results further showed that four domains of servant leadership namely empowerment, humility, stewardship and authenticity were negatively and significantly related to employee turnover intentions. The dimensions of accountability and courage were not significantly related to turnover intentions. The implication of these results is that the servant leadership behaviors including empowerment, humility, stewardship and authenticity may be relevant for ameliorating employee turnover intentions in the sub-Saharan Africa.

## Practical implications

This study shows that the servant leadership scale is an effective tool to use in the SSA to enhance effective leadership in the healthcare sector. Findings of this validation study can be used to inform management in the design and implementation of culturally contextualized leadership development programs in SSA. The findings are indicative of the relevance of servant leadership domains in informing culture definition and development programs in workplaces in SSA.

Furthermore, SSA countries also have the need to on the one hand to minimize costs while maintaining high standards of patient care. The study shows that by embracing servant leadership behaviors, employees are less reluctant to leave the organization, which results in reducing costs of recruiting and training new employees. Moreover, servant leadership increases the sense of organizational justice which has been shown to increase employee wellbeing and job satisfaction (Farrington and Lillah, 2019) which ultimately leads to better patient care.

## Limitations and suggestions for future research

As with all other research of this nature, this study is not without its limitations. The results of this study are constrained by its cross-sectional design with data obtained at one point in time. This prohibits a definitive causal inference on the relationship between the variables (Jiao *et al.*, 2011).

As with much of the leadership research, raters were required to provide an evaluation of the immediate supervisor based on their recollection of the focal behavior of their supervisor. As such, we cannot be sure that raters are referring to specific instances of such behaviors or to an implicit evaluation of their leader (Michel *et al.*, 2011).

The current research provides a foundation for further research into servant leadership especially in the healthcare sector in sub-Sahara Africa. Envisaged future research should use alternative research designs such as longitudinal, ethnographic and experimental designs. Berson (1999) has recommended integrating both quantitative and qualitative methods in the form of triangulation to obtain a more comprehensive and valid assessment of leadership (Antonakis *et al.*, 2003).

Furthermore, empowerment, humility, stewardship and authenticity appear to be the most highly correlated items to both organizational justice and turnover intentions, which could suggest a need to further explore if these four factors are the most outstanding servant leadership domains.

It would be important to also validate the SLS scale within other contexts in the sub-Saharan region, to verify whether “standing back” and “forgiveness” do not meet significance again. If this was the case, it would be important to understand if there are cultural aspects that are influencing these results.

Future research should benefit from the inclusion of alternative outcome variables such as job satisfaction, organizational commitment, employee engagement, work alienation etc. as well as consideration of other emerging leadership approaches in healthcare such as reflective leadership. The inclusion of alternative outcome variables will enable the assessment of the effectiveness of various leadership approaches beyond a limited number of variables. Emerging leadership approaches should be included so that no opportunity is lost in the search for effective leadership.
